# Tunable optical analog to electromagnetically induced transparency in graphene-ring resonators system

**DOI:** 10.1038/srep38891

**Published:** 2016-12-12

**Authors:** Yonghua Wang, Chenyang Xue, Zengxing Zhang, Hua Zheng, Wendong Zhang, Shubin Yan

**Affiliations:** 1 Key Laboratory of Instrumentation Science & Dynamic Measurement (North University of China), Ministry of Education, Taiyuan 030051, Shanxi Province, China

## Abstract

The analogue of electromagnetically induced transparency in optical ways has shown great potential in optical delay and quantum-information technology due to its flexible design and easy implementation. The chief drawback for these devices is the bad tunability. Here we demonstrate a tunable optical transparency system formed by graphene-silicon microrings which could control the transparent window by electro-optical means. The device consists of cascaded coupled ring resonators and a graphene/graphene capacitor which integrated on one of the rings. By tuning the Fermi level of the graphene sheets, we can modulate the round-trip ring loss so that the transparency window can be dynamically tuned. The results provide a new method for the manipulation and transmission of light in highly integrated optical circuits and quantum information storage devices.

Since it has been proposed, the electromagnetically-induced transparency (EIT) effect has shown great potential in various applications, including slow light control, quantum information processing and sensing technology^1^,^2^,^3^. But the realization of EIT in atomic systems is a difficult task since some restrictions must be strictly fulfilled. In recent years, many studies have demonstrated that the EIT effect can be mimicked in classical optical coupled resonators due to its flexible design and easy implementation^4^,^5^,^6^,^7^,^8^,^9^,^10^,^11^. The optical EIT-like effect has been experimentally realized in many optical structures including coupled fiber systems^4^, coupled fused-silica spheres^5^, optical parameter oscillator cavities^6^, and the on-chip systems such as coupled silicon microring resonators^7^,^8^,^9^, microdisk resonators^10^, and photonic crystal microcavities^11^. However, most of these structures are immobile or only can be tuned by mechanical alignment^12^. It is difficult to realize the dynamic control of the transparency window without changing their geometrical parameters, which limits the practical application of EIT-like effect.

Graphene, a new material that has been available for only a decade, has generated great interest due to its exceptional properties such as high carrier mobility^13^, broadband absorption^14^,^15^, zero-band gap and tunable Fermi level^16^,^17^,^18^. In particular, the remarkable optical properties^19^ have made graphene a promising material for fast and broadband electro-optic devices such as photodetectors^20^,^21^ and modulators^22^,^23^,^24^,^25^. In these graphene integrated modulators, interband transitions and optical constants can be tuned through electrical gating. While the EIT-like effect in cascaded resonators has a specific relation with the optical constants of the resonators, confirming that graphene also has an excellent ability in controlling the EIT-like effect. Furthermore, all this structures are COMS-compatible.

In this work, we propose and experimentally demonstrate the EIT-like effect on graphene-silicon cascaded micoring resonators in which the transparency peak can be dynamically tuned via electrically control. By applying increasing voltage to the graphene/graphene capacitor which has been covered on one of the ring waveguides, regular changes of the EIT-like spectrum have been observed.

## Results

### Device design and fabrication

**Figure 1:**
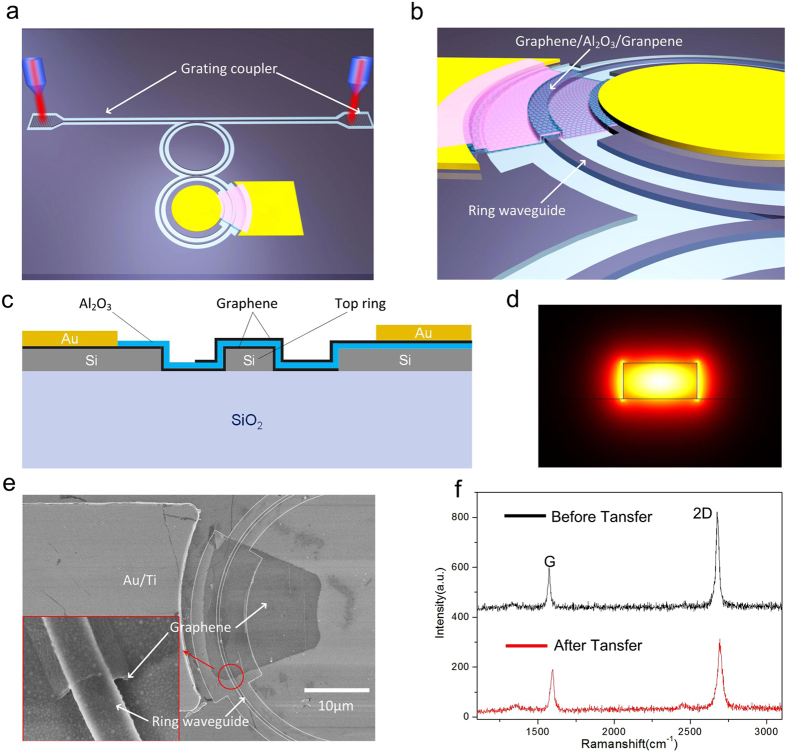
Device design. (**a**) Over-all structure of the cascaded rings integrated with the graphene layers. (**b**) Functional region of the double graphene layers covering on the top ring. (**c**) Cross-section of the top ring covered with the bottom and top graphene layers separated by 20 nm Al_2_O_3_. (**d**) The corresponding TE mode-field profile of the 450 × 220 nm waveguide. (**e**) Electron microscopy of the functional region and the top graphene layer before the second Ti/Au contacts and the SiO_2_ upper cladding were deposited (**f**) Raman spectrum of the graphene sheet before transfer(black curve) and after transfer(red curve) to the device. After transfer, the G peak is at 1596 cm^−1^ and 2D peak is at 2692 cm^−1^.

The structure of our device is depicted in Fig. 1a and b, showing the two cascaded ring resonators coupled with the bus waveguide. When light is coupled into the ring from the bus waveguide due to the evanescent field, the coherent interference between the properly designed coupled resonators will generate the EIT-like effect. In order to tune the transparency peak, two graphene layers which separated by an oxide are placed on the top ring. The sandwich structure formed by two graphene layers makes up a simple parallel capacitor model. The cross-sectional structure is shown in Fig. 1c. When a gate voltage is added, the two graphene layers are doped, one with holes and the other with electrons at the same doping level. causing a significant shift in Fermi energy (*E*_F_), which will in turn alter the rate of interband transitions and, subsequently, the optical transitions^18^.

The device was fabricated on commercial SOI wafers, having a silicon thickness of 220 nm and a buried-oxide thickness of 3 μm. The bus waveguide and the two cascaded ring resonators were fully etched, with the depth of 220 nm and a width of 450 nm, to guide single-mode transverse electric (TE) light (Fig. 1d). The radii of the two ring resonators were designed to be 20 μm. Two gaps, between the two rings and between the ring and bus waveguide, were designed to have the same length, approximately equal to 90 nm approximately which was found to be the optimum coupling distance for our device. At both ends of the bus waveguide, a pair of grating couplers was fabricated with a 600 nm period, in order to couple light in and out of the devices. Before transferring graphene layer, a 5 nm atomic layer deposited (ALD) Al_2_O_3_ was employed to prevent potential carrier injections from the bottom graphene layer into the silicon^23^. A graphene/graphene capacitor was fabricated above the top ring resonator. The capacitor consists of two sheets of monolayer graphene grown by means of chemical vapour deposition (CVD) on copper foil and wet-transferred on our silicon device (see method). The electron microscopy of the functional region and the top graphene layer before the second Ti/Au contacts and the SiO_2_ upper cladding were deposited are shown in Fig. 1e and we can see a high quality coverage of graphene on the silicon substrate and the waveguide. Raman spectrum of the graphene sheet before and after transfer to the device is shown in Fig. 1f. The oxide between two graphene layers was 20 nm Al_2_O_3_, obtained using the same ALD method. To apply voltage to the capacitor Ti/Au (10/100 nm) contacts were used for both layers of graphene by electron-beam evaporation. We then provided 1 μm-thick SiO_2_ upper cladding deposited by plasma-enhanced chemical vapor deposition to avoid the loss caused by metal contact.

### Theoretical description of the tunable optical transparency mechanism

**Figure 2:**
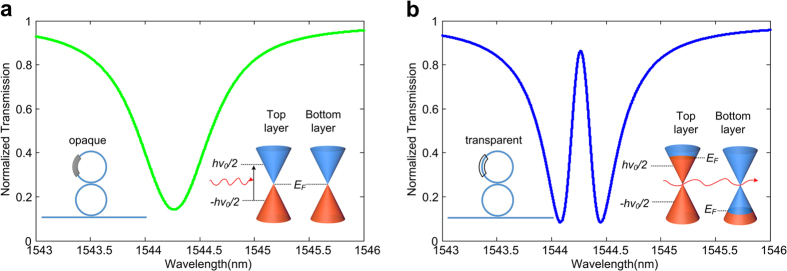
Relationship between the Fermi level and the EIT-like effect. (**a**) At low bias voltage, the Fermi level is close to the Dirac point, light transmission in the top ring is absorbed by the graphene layers and EIT-like effect does not occur. (**b**) At large bias voltage, no interband transition occurs, the graphene layers are transparent and the EIT-like effect is significant.

When a bias voltage *V*_b_ is added, the change of the carrier concentrations in graphene can be expressed as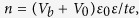 where *V*_0_ is the offset voltage caused by natural doping, *ε*_0_ and *ε* are the permittivities of free space and of Al_2_O_3_, respectively, and *t* is the thickness of the Al_2_O_3_ between the two graphene layers, *e* is charge of the electron. Thus, the Fermi level of graphene will be shift as 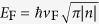, where *ħ* is the Plank constant divided by 2π and *v*_F_ is the Fermi velocity. When the bias voltage dependent Fermi level *E*_F_(*V*_b_) is close to the Dirac point, interband absorption will occur as electrons are excited by the incoming photons (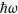). When this occurs, the graphene layers will not be transparent^22^. On the other hand, when *E*_F_(*V*_b_) is greater than half of the photon energy of incident light (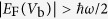), all electron states are filled up in graphene and no interband transition is allowed due to the Pauli blocking. In this condition, graphene layers become transparent. As a consequence of the absorption change in the graphene layers, the ring waveguide under them will experience a significant change in round-trip loss. According to the mechanism of the EIT-like effect in optic resonators, the loss of the top ring waveguide has a specific influence on the transparency window (Supplementary Note 1). The cascaded optical transparency window varies with the attenuation factor coefficient of the top ring. The increase of the attenuation entails a decrease of the round trip loss, which has a direct correlation with the absorption of graphene (Supplementary Fig. 1b). The relationship between the Fermi level and the EIT-like effect can be illustrated as in Fig. 2. When bias voltage is low, both graphene layers are lightly doped (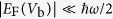) and thus opaque, inducing a high loss in the ring waveguide and the EIT-like effect will not occur if the graphene lays has enough coverage (Fig. 2a). When bias voltage is large, both graphene layers are heavily doped, by electrons and holes respectively (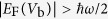), and thus transparent. As a consequence the ring waveguide has low loss and the EIT-like effect will occur (Fig. 2b). Therefore, according to this theory, the EIT effect can be dynamic tuned by changing the bias voltage without changing the geometrical parameters of the device.

### Device measurement

**Figure 3:**
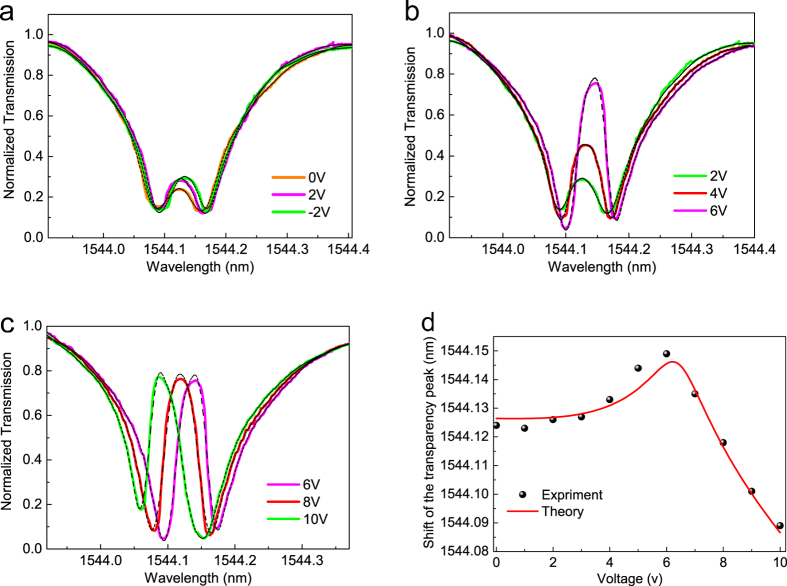
EIT-like effect controlled by bias voltage. (**a**) Transparency window at the d.c. voltage of 0 V (orange curve), 2 V (magenta curve) and −2 V (green curve). The curves at 2 V and −2 V are not coincident due to the different environments for the two graphene layers. (**b**) Increasingly apparent transparency peak and the red shift with the increase of the voltage from 2 V (green curve) to 4 V (red curve), then 6 V (magenta curve). The broadened absorption of graphene leads to a gradual change of transparency peak. (**c**) Interband transitions are blocked when voltage is larger than 6 V so the peak value no longer increases and only the blue shift changes is observed with the increasing voltage. (**d**) Experimental and theoretical shift of the transparency peak with the variation of the voltage. The red and blue shifts of the transparency peak are due to an increase and subsequent decrease of 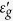 in graphene.

The tunability of the graphene-ring resonator based EIT-like device is investigated by applying different bias voltages. For zero bias voltage, Fermi level is closed to the Dirac point and the top ring has relative high loss and the EIT-like effect is consequently weak (Fig. 3a), corresponding to the condition shown in Fig. 2a. We can notice that there is still a small transparency peak at 0 V bias, indicating that the EIT-like optical interference still exist between the cascaded rings at this loss condition. Figure 3a also shows the transparency windows in 2 V and −2 V bias voltages in which we can see that the 2 V and −2 V curves are not coincident. Having designed the device, it is known the two graphene layers covered on the top ring are equivalent and therefore should have the same response to bias voltages having the same absolute value. We can ascribe this difference to the different processing technologies adopted for Al_2_O_3_ and SiO_2_, which modify the surrounding environments of the two graphene layers. The top layer is in contact with Al_2_O_3_ on one side and with SiO_2_ on the other; the bottom layer is instead sandwiched between two Al_2_O_3_ layers^23^. With the bias voltage increasing from 2 to 6 V, the transparency peak become significant, as shown in Fig. 3b. This is a direct consequence of blocked graphene optical absorption with the increasing doping level, which leads to a lower loss. Ideally, the EIT-like transparency peak should emerge instantaneously when the Fermi level is increased above the 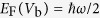 threshold (or lowered below 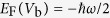) as shown in Fig. 2. However, the loss in the top ring is essentially determined by the imaginary part of the effective index (*n*_*eff*_) of the graphene-silicon waveguide, which is mainly decided by the Fermi level-dependent dielectric constant (*ε*_g_) of graphene. The dielectric constant *ε*_g_ can be obtained through 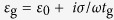
26, where *ω* is the frequency, *t*_g_ is the thickness of the graphene layer, and *σ* is the optical conductivity of graphene. The overall mode in the graphene-silicon waveguide displays a Gaussian distribution. The graphene layers only capture very few portions of the fields and the fields outside the graphene layer cannot feel *ε*_g_ variations^27^. As a consequence, the transmission in the graphene-silicon waveguide varies gently around the threshold 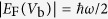. In other words, the interband transition induced absorption is broadened and the transparency window will have a gradual change with the shift of the Fermi level. This is one reason why the EIT-like transparency peak varies gently with the increase of the bias voltage. While the other one which associate with the interband relaxation time will be described in the following paragraphs. With the voltage increasing from 6 to 10 V, the increase of amplitude of the transparency peak is no longer significant, as shown in Fig. 3c. This indicates that the loss induced by graphene has been lowered to a minimum due to the Pauli blocking and the loss is now mainly attributable to the silicon waveguide. Meanwhile, the wavelength spectrum has a red shift with the voltage increasing from 2 to 6 V (Fig. 3b), and a blue shift from 6 to 10 V (Fig. 3c). This can be attributed to the real part of the dielectric constant (*ε*_g_′) of graphene which changes with the voltage-dependent Fermi level and is similar to the resonance frequency shifts observed in optical plasmon resonance with graphene^28^, as we describe below.

The real and imaginary parts of the complex dielectric constant, *ε*_g_′ and *ε*_g_″, of graphene can be obtained from its optical conductivity (*σ*), which can be derived from the Kubo formula in a complex form consisting of interband and intraband contributions^29^,^30^


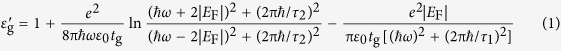



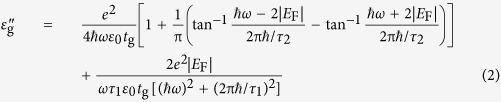


where 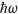 is the photon energy of the incident light, set to 0.805 eV at the region of incident light we choosed (i.e., at the wavelength of 1544 nm). 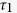 is the relaxation time associated with carrier-carrier intraband collisions and phonon emission and *τ*_2_ is the relaxation time associated with electron interband relaxation and cooling of hot phonons^31^,^32^,^33^,^34^. The term 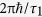 can be set to zero because the the relaxation time associated with intraband transition usually in the order of the hundreds femtosecond^31^ and thus it has little effect on the dielectric constant. Here we set 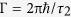, which can be treated as the interband transition broadening^28^,^35^. The value of *τ*_2_ has been measured on a picosecond scale^33^,^34^, but the value of Γ is usually enlarged owing to the defects in graphene. This is another reason why the EIT-like transparency peak varies gently with the increase of the bias voltage (Supplementary Fig. 2b). Equation 2 shows that 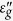 experiences a step-like decrease when the Fermi level is above the threshold 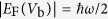 indicating that the interband transitions are blocked. This is because the overall mode index in the graphene-silicon waveguide *n*_*eff*_ is proportional to graphene’s dielectric constant *ε*_g_ in both real and imaginary parts^27^. The imaginary part of the index Im(*n*_*eff*_) represents the loss in the waveguide and it is the same for 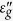. 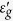 shows the same trend with the real part of the index Re(*n*_*eff*_) which has a proportional relationship with the wavelength shift of the transmission curve (Supplementary Note 1). Therefore, the red and blue shift of the transparency peak can be well explained by the character of 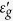. When 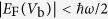, 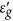 increases with the increasing of *E*_*F*_(*V*_*b*_) and so does Re(*n*_*eff*_), so the transparency curve has a red shift (Fig. 3b). When 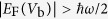, 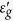 decreases with the increasing of *E*_*F*_(*V*_*b*_) and so does Re(*n*_*eff*_), so the transparency curve has a blue shift (Fig. 3c). Figure 3d shows the experimental shift of the transparency peak with the variation of the bias voltage. The theoretical curve simulated by 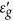 agrees well with the experimental result. In the course of the theoretical simulation, *E*_*F*_ + 0.24 eV was used instead of *E*_*F*_ in equation 2 because the natural doping in graphene has a shift in the Fermi level, as will be described below.

From equation 1, it is possible to notice that 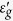 peaks around *E*_*F*_ = 0.4 eV (i.e. the threshold 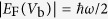) and the corresponding bias voltage of 3.9 V can be calculated using the relationship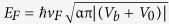, where 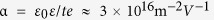 as estimated from a parallel-plate capacitor model of our device. From the Raman spectroscopy (Fig. 1f) we can estimate a p-type doping approximately equal to 4.4 × 10^12^cm^−2^ (Supplementary Note 2) and thus the corresponding zero bias voltage Fermi level *E*_*F*_ = 0.24 eV and the offset voltage *V*_0_ = 1.46 V can be obtained. As a result, the bias voltage relevant to the transmission threshold 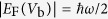 can be calculated to be equal to 5.36 V. However, either the saturate of the peak value (Fig. 3c) or the bending of the transparency peak shift (Fig. 3d) indicate that the threshold voltage is around 6 V. The small difference of the threshold voltage can be attributed to the quantum capacitance of graphene which has been measured on a microfarad per square centimeter scale^36^.

## Discussion

We have experimentally demonstrated, for the first time to our knowledge, the tunable optical analog to electromagnetically induced transparency on chip by integrating monolayer graphene with the cascaded microring resonators. The relationship between the EIT-like effect and the voltage dependent Fermi level of graphene has been investigated through both theoretical studies and experimental measurements. The transmission spectra reveal that the structure possesses a prominent transparency peak at the threshold voltage of 6 V, indicating that the Fermi level of graphene has reached half of the photon energy (i.e. 0.4 eV) at this voltage. Furthermore, the red and blue shifts of the transparency window have been well explained in terms of the real part of the dielectric constant of graphene. Our results will provide important guidelines for the design and use of tunable optical EIT devices which have promising applications in slow light, quantum information processing and sensing technology, and provide a new method for the manipulation and transmission of light in highly integrated optical circuits. Furthermore, compare with the mechanical tuning of the EIT-like effect, the tuning speed of the device could be as high as the one obtained with modulators^22^,^23^,^24^,^25^ which utilize the graphene in a similar way. This feature may be useful in some special cases of fast on-chip optical communications.

## Methods

### Device fabrication

The cascaded ring waveguides were fabricated in the SOI wafer with a silicon thickness of 220 nm and a buried-oxide thickness of 3 μm. E-beam lithography (EBL) and inductively coupled plasma (ICP) etching were used to fabricate the 220 × 450 nm waveguides and the 300 × 300 nm gratings. The region for graphene transfer (the etching era for the top ring) was broadened to obtain a good coverage of graphene. Before the transfer of the bottom graphene, 5 nm isolation layer deposited (ALD) Al_2_O_3_ was used to prevent potential carrier injections from the bottom graphene layer into the silicon. 200 nm thick polymethyl methacrylate (PMMA) was spin-coated onto the graphene sheet which was grown by chemical vapour deposition (CVD) on Cu film and baked at 200 °C for 1 min. The copper foil was then removed using FeCl_3_ solution and the graphene sheet, coated with PMMA, was transferred onto the device. Finally, the PMMA was removed using acetone and the residue was rinsed with isopropanol to obtain a clean graphene surface. To remove the unwanted part of graphene, the deep-ultraviolet lithography and oxygen plasma were used. Before deposing the sandwich 20 nm Al_2_O_3_, 10 nm Ti and 50 nm Au were deposited by electron-beam evaporation and patterned by a lift-off process. This was done to protect the bottom graphene from the sandwich Al_2_O_3_ etching process for electric contacting. Owing to the hydrophobic nature of the graphene basal plane, it is difficult to depose Al_2_O_3_ directly on it. For this reason, before deposing Al_2_O_3_, a 1 nm aluminum layer was evaporated using electron-beam evaporation and immediately oxidized into Al_2_O_3_ in ambient air^23^. Using the 1 nm Al_2_O_3_ as the seed layer, the sandwich Al_2_O_3_ was then deposited on the device. In order to contact the electrode with the bottom graphene layer, which was fully covered by Al_2_O_3_, reactive ion etching (RIE) was used to remove a patch of Al_2_O_3_ from the Ti/Au (10/50 nm) which had been previously deposed. The transfer procedures used for top graphene layer was the same with the bottom one. The final device was then covered with 1 μm-thick SiO_2_ deposited by plasma-enhanced chemical vapor deposition (PECVD) and drilled by RIE to contact the two graphene layers with the electrode.

## Additional Information

**How to cite this article**: Wang, Y. *et al*. Tunable optical analog to electromagnetically induced transparency in graphene-ring resonators system. *Sci. Rep.* **6**, 38891; doi: 10.1038/srep38891 (2016).

**Publisher's note:** Springer Nature remains neutral with regard to jurisdictional claims in published maps and institutional affiliations.

## Supplementary Material

Supplementary Information
