# Supplementary material for: Tunable optical analog to electromagnetically induced transparency in graphene-ring resonators system
Source: Sci Rep. 2016 Dec 12;6:38891. doi: 10.1038/srep38891 (PMC5151055; doi:10.1038/srep38891)
Supplement: Supplementary Information [file srep38891-s1.pdf]

Tunable optical analog to electromagnetically induced transparency in graphene-ring resonators system

Yonghua Wang[1], Chenyang Xue[1,*], Zengxing Zhang[1], Hua Zheng[1], Wendong Zhang[1], Shubin Yan[1]

Supplementary Figures

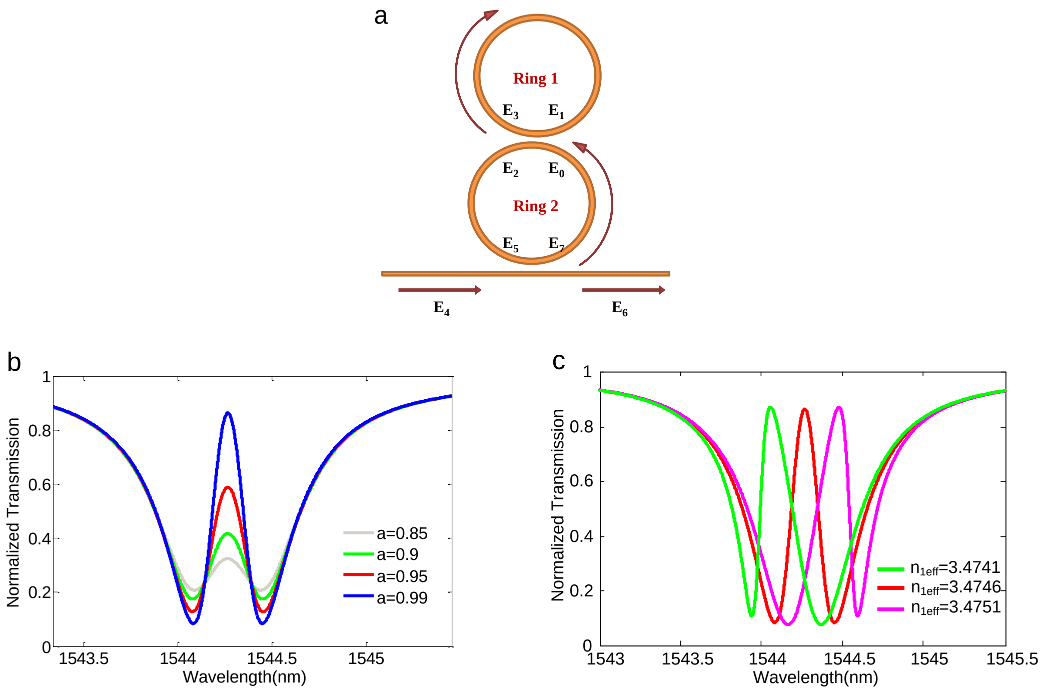

Supplementary Figure 1 | Theory for optical analog to electromagnetically induced transparency in cascaded ring resonators. (a) Schematic of the cascaded ring resonators and the incoming and outgoing electric fields at the coupling region. **(b)** The cascaded optical transparency window varies with the attenuation factor coefficient of ring 1. The increase of the attenuation entails a decrease of the round trip loss, which has a direct correlation with the absorption of graphene. **(c)** The impact effective index of ring 1 on transparency peak. The increase of effective index cause a red shift and conversely a blue shift.

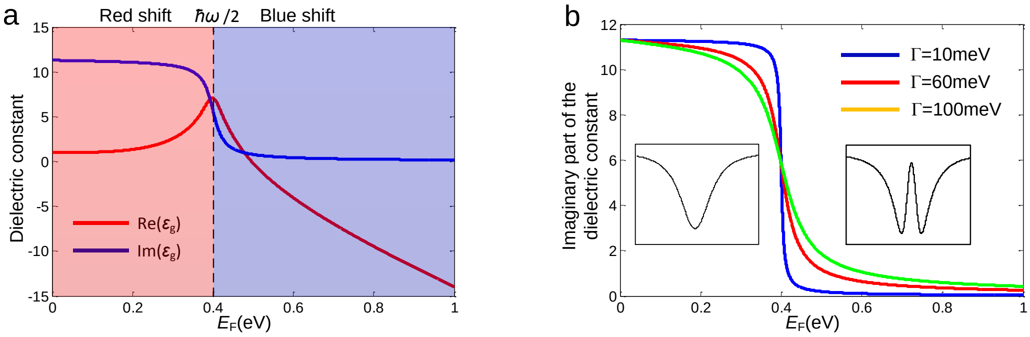

Supplementary Figure 2 | Relationship between the transparency peak with real part and

imaginary part of dielectric constant. (a) The change in real part and imaginary part of dielectric constant with the Fermi level. The Re(ε_g) experiences a step-like decrease and the Im(ε_g) shows a tendency of first ascending and then descending when the Fermi level is above $\hbar\omega/2$. For Re(ε_g), the line cut by $\hbar\omega/2$ is the divide of red shift and blue shift of the transparency peak while for Im(ε_g), the cutting line is the threshold of the interband transition blocking. **(b)** The change in imaginary part of dielectric constant with the interband transition broadening Γ. With the increase of the broadening, the Im(ε_g) becomes gentle at the threshold $\hbar\omega/2$, indicating the absorption is not a sudden change at the threshold and thus the transparency peak experiences a gradual change with the voltage.

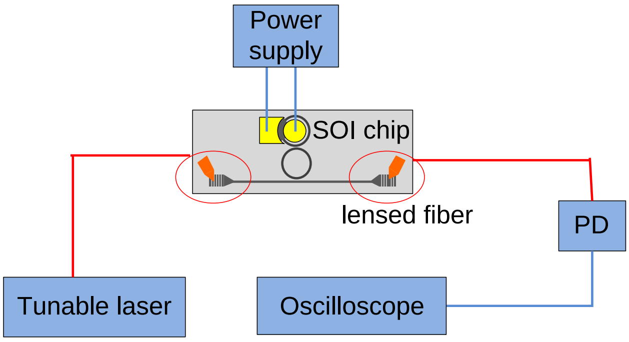

Supplementary Figure 3 | Device measurement setup. The generation of the transparency curve is obtained by sweeping the frequency of the tunable laser (New Focus TLB-6728-P). Light export from the tunable laser source is polarization-tuned by a polarization controller and injected into the grating coupler using a 75 °lensed fiber and aligning by a precision translation Stage (New Focus M-461-XYZ-M). The output light is detected by a photoelectric detector (New Focus 1811) and display on the digital oscilloscope (Tektronix MDO3054).

Supplementary Note

Supplementary Note 1 | Theory for optical analog to electromagnetically induced transparency in cascaded ring resonators.

The couplings between the resonators and the bus waveguide are obtained through the evanescent field. The reflected and transmitted electric fields (Supplementary Fig. 1a), respectively, for the unidirectional coupling of light to the ring1 resonator can be express as

$$E_2=r_1E_0+it_1E_1 \quad (1)$$

$$E_3=it_1E_0+r_1E_1 \quad (2)$$

where r_1 and t_1 are the reflectivity and transmittivity of the coupler and are correlated by $t_1{}^2+r_1{}^2=1$. The propagation along ring1 can be express as

$$E_1=a_1\exp(i\delta_1)E_3 \quad (3)$$

where $a_1=\exp(-\alpha_1L_1/2)$ and δ_1 are the attenuation factor coefficient and the single pass phase shift of ring 1, respectively. Here, α and L_1 are the absorption coefficient and the length of ring 1, respectively. Substituting Eq. (3) into Eq. (1) and Eq. (2), we can obtain the ratio

$$\frac{E_2}{E_0}=\frac{r_1\text{-}a_1\exp(i\delta_1)}{1\text{-}r_1a_1\exp(i\delta_1)}\equiv\tilde{\tau}_1 \quad (4)$$

Using the same method, we can obtain the relationship between the input and output electric fields

$$\frac{E_6}{E_4}=\frac{r_2\text{-}a_2\exp(i\delta_2)}{1\text{-}r_2a_2\tau_1\exp(i\delta_2)}\equiv\tilde{\tau}_2 \tag{5}$$

From Eq. (4), the effective phase shift for ring 1 can be obtained

$$\tilde{\delta}_1^{(\text{eff})}\equiv\arg(\tilde{\tau}_1)=\pi+\delta_1+\arg[\frac{a_1\text{-}r_1\exp(i\delta_1)}{1\text{-}r_1a_1\exp(i\delta_1)}] \tag{6}$$

Therefore, the absorption of the whole system can be express as the configuration analog to the EIT in a Λ-type atomic three-level system (Reference 1)

$$\tilde{A}=\frac{\tilde{A}_2^{(\text{env})}}{1+\tilde{F}_2\sin^2(\frac{\tilde{\delta}_1^{(\text{eff})}+\delta_2}{2})} \tag{7}$$

where

$$\tilde{A}_2^{(\text{env})}\equiv\frac{(1\text{-}r_2)^2\text{-}(1\text{-}a_2^2|\tilde{\tau}_1|^2)}{1\text{-}r_2a_2|\tilde{\tau}_1|^2} \tag{8}$$

is an envelope function,

$$\tilde{F}_2\equiv\frac{4r_2a_2|\tilde{\tau}_1|}{(1\text{-}r_2a_2|\tilde{\tau}_1|)^2} \tag{9}$$

is a function related to finesse.

In our device, the tuning of the EIT-like effect is essentially consequence of changing the loss of ring 1 by graphene (i.e. changing attenuation factor coefficient a_1). The change of a_1 will lead to the change of the transparency window, as shown in Supplementary Fig. 1b. With the increase of a_1, the transparency peak become increasingly significant, indicating the EIT effect is getting stronger.

The relationship between the single-pass phase shift and the effective index of ring 1 can be expressed as $\delta_1=2\pi n_{\text{eff1}}L/\lambda$. We can find that the phase shift is in direct proportion to the effective index. Therefore, the shift of the transparency peak can be attributed to the effective index change of ring 1. Supplementary Fig. 1c shows the shift of the transparency peak with different effective index, which agrees well with our result.

Supplementary Note 2 | The Fermi level of graphene calculated from the G band phonon energy of the Raman spectrum.

Owing to the doping of the external environment, the Fermi level of graphene usually deflects from the Dirac point. The Raman spectrum shows the G peak is at 1596 cm^{-1} and 2D peak is at 2692cm^{-1}, indicating the Fermi level is blow the Dirac point. The p-type doping of graphene can be attribute to the external environment including hydrone, the oxide base and the residual polymethyl methacrylate (PMMA) after the transfer of the sheet. Furthermore, the G band phonon energy has a good quantitative relation with the Fermi level (Supplementary References 2)

$$\hbar\omega_G\text{-}\hbar\omega_G^0=\frac{A_{\text{uc}}D^2}{2\pi\hbar\omega_G M v_F^2}\left[|E_F|+\frac{\hbar\omega_G}{4}\ln\left|\frac{2|E_F|\text{-}\hbar\omega_G}{2|E_F|+\hbar\omega_G}\right|\right] \tag{10}$$

where ω_G^0 is ω_G at the Dirac point, A_{uc} is the area of the graphene unit cell, *M* is the carbon atom mass, and *D* is the electron-phonon coupling strength. Combining Eq. 10 with $E_F=\hbar v_F\sqrt{\alpha\pi|n|}$, we can obtain the carrier concentration n=4.4×$10^{12}$$cm^{-2}$.

Supplementary References:

1. Smith, David D. *et al*. Coupled-resonator-induced transparency. *Phys. Rev. A* **69**, 666-670 (2004).

2. Yan, J. et al. Electric field effect tuning of electron-phonon coupling in graphene. *Phys. Rev. Lett.*, **98**, 166802 (2007).
